# Targeted ‘knock out’ therapy with a combination antimicrobial regimen restores treatment options in the management of extensively drug-resistant carbapenemase-producing organisms

**DOI:** 10.1099/jmm.0.002007

**Published:** 2025-04-22

**Authors:** Saied Ali, Orla Donoghue, Sinead McDermott

**Affiliations:** 1 Department of Clinical Microbiology, St. Vincent’s University Hospital, Dublin, Ireland; 2 Royal College of Surgeons in Ireland, Dublin, Ireland

**Keywords:** antimicrobials, antimicrobial resistance, antimicrobial stewardship, aztreonam, carbapenems, cefiderocol, ceftazidime-avibactam

## Abstract

**Introduction.** Cefiderocol (FDC) and the combination of ceftazidime-avibactam and aztreonam (CZA+ATM) are emerging therapeutic options to combat carbapenemase-producing organisms (CPOs) that exhibit resistance due to multiple *β*-lactamases.

**Hypothesis/Gap Statement.** Molecular diagnostics and specialized antimicrobial susceptibility testing (AST) are infrequently available in most clinical laboratories, and outputs from reference laboratories are not always timely. Practical methods must be explored to provide meaningful advice to treat infections due to CPOs in real time.

**Aim.** To evaluate the *in vitro* efficacy of FDC and CZA+ATM against CPOs and to compare colistin (CST) MICs obtained locally with those from the reference laboratory.

**Methodology.** CPOs isolated from 2017 to 2023 inclusive were retrieved. AST for FDC was performed using disc diffusion, CZA and ATM individually by E-tests and the E-test superposition method for the combination CZA+ATM. CST AST was performed locally using the VITEK2 system, and MICs were compared with those attained from the reference laboratory where manual broth microdilution is performed.

**Results.** Fifty-eight CPOs were analysed. OXA-48 was the most frequently detected carbapenemase (37.9%, *n*=22). Co-existing *β*-lactamases of Ambler classes A and C were present for 79.3% of CPOs (*n*=46). Twenty-nine isolates (50%) were found to be susceptible to FDC. Fifty-seven isolates (98.3%) were susceptible to CST according to the VITEK2, compared to 44 of 47 tested isolates (93.6%) by the reference broth microdilution. Essential agreement was found to be 78.7%, and categorical agreement was 91.5% with one major error and three very major errors (VMEs) reported. CZA+ATM was tested against 26 CPOs, all of which harboured metallo-*β*-lactamases. Synergy was detected for all except one isolate where additivity was noted. Of the 32 isolates where combination therapy was not assessed, 29 (90.6%) possessed serine-*β*-lactamases and were susceptible to CZA monotherapy, whilst three (9.4%) possessed an isolated metallo-*β*-lactamase and were susceptible to ATM monotherapy.

**Conclusions.** FDC appears to perform favourably against CPOs harbouring serine-*β*-lactamases, but not metallo-*β*-lactamases. The VITEK2 may provide presumptive categorical information for CST susceptibility, but MICs must be confirmed by broth microdilution as VMEs can lead to treatment failures. Moreover, our study confirms potent *in vitro* activity of CZA+ATM against CPOs expressing multiple *β*-lactamases.

## Introduction

In the Republic of Ireland, invasive infections due to carbapenem-resistant Enterobacterales (CRE) were made notifiable in 2011, accompanied by a voluntary enhanced surveillance system for all CRE isolates [1]. In 2013, in response to rising multi-drug resistant *Klebsiella pneumoniae* (MDRKP) infections, including carbapenem-resistant strains, a national MDRKP outbreak control team was established, and a mandatory enhanced surveillance scheme for all MDRKP isolates was introduced in January 2014 [1,2]. By 2016, MDRKP was reported in 88% of Irish hospitals, with cases also identified in primary and residential care, indicating widespread dissemination. Of particular concern was a 195% increase in carbapenemase-producing MDRKP in 2016 compared to 2015 [2], prompting the establishment of the mandatory carbapenemase-producing Enterobacterales (CPE) enhanced surveillance scheme in January 2017 and the declaration of a national public health emergency to address this worrying rise in detections of carbapenemase-producing organisms (CPOs) in acute hospitals [1,3, 4].

In Ireland, the number of patients with newly confirmed CPE increased from 50 in 2013 to 433 in 2017 and 1,096 in 2023. Additionally, outbreaks driven by CPE have risen from 26 in 2022 to 33 in 2023 [1]. This trend was also seen across the European Union, with an estimated total incidence of 3.97 per 100,000 population of carbapenem-resistant *K. pneumoniae* bloodstream infections – a 57.5% increase in 2023 compared to 2019 [5,6]. Despite heightened awareness and numerous infection prevention and control (IPC) and public health interventions [7], cases continue to rise in the context of globalization, resource limitations and architectural inefficiencies within healthcare systems [8,13].

Since then, the predominant circulating carbapenemase strain in Ireland has been OXA-48, with ≥70% of reported isolates from the National Carbapenemase-Producing Enterobacterales Reference Laboratory Service (NCPERLS), based at Galway University Hospital, identified as OXA-48 producers. However, case detections of other carbapenemases, such as *K. pneumoniae* carbapenemase (KPC) and New Delhi metallo-*β*-lactamase (MBL) (NDM), have been increasing [1,14].

A meta-analysis reported up to 44% mortality associated with CPO infections [15], with similar rates of 44%, 53.2% and 56.7% observed in multicentre studies from the UK [16], South Korea [17,18] and India [19]. These studies included diverse patient populations with varying comorbidities, and whilst not statistically significant, patients with higher comorbidity burdens were more likely to succumb to infection. This high mortality is largely thought to be driven by the limited availability of effective therapeutic options amongst traditionally used antimicrobial agents, which include potentially toxic drugs like polymyxins.

Ambler class B carbapenemases, such as the NDM, Verona integron-encoded MBL (VIM) and integron-associated MBL (IMP), are particularly drug-resistant and are an enormous treatment challenge in the clinical setting [20,21]. CPOs frequently harbour co-existing resistance mechanisms, such as extended-spectrum beta-lactamases (ESBLs), which further complicate and restrict antimicrobial treatment selection [22,25].

Cefiderocol (FDC) is a novel siderophore cephalosporin with potent activity against a range of Gram-negative organisms, including CPOs. Its molecular structure with various side chains and its catechol moiety maintains stability against hydrolysis by both serine-*β*-lactamases (SBLs) and MBLs, thus providing a valuable alternative treatment option [26,28].

The combination of ceftazidime (CTZ)-avibactam and aztreonam (CZA+ATM) is also an emerging therapeutic option to combat CPOs expressing resistance via multiple *β*-lactamases within Ambler classes A, B, C and D. Whilst CZA possesses activity against organisms producing SBLs within Ambler classes A, C and D due to its *β*-lactamase inhibitor – avibactam (AVI) – it is inactive against those producing MBLs from the VIM, IMP or NDM groups within Ambler class B. ATM is not hydrolysed by MBLs, but it is readily degraded by most SBLs which are often carried concomitantly, thereby making it ineffective as monotherapy for infections caused by these organisms. By inhibiting co-existing SBLs with AVI, through the administration of CZA, the activity of ATM against MBL-producing organisms is restored and has been experimentally validated [29,31].

Colistin (CST), a polymyxin antimicrobial, belongs to the class of lipopeptides. It exerts its bactericidal effect primarily by disrupting the bacterial cell membrane, through binding to lipopolysaccharides in Gram-negative bacteria [32]. CST antimicrobial susceptibility testing (AST) is pivotal in guiding effective antimicrobial use, especially when CST serves as a last-resort treatment. Whilst automated systems like VITEK2 offer convenience, they often lack precision due to limitations in accurately detecting MICs. Additionally, the automated nature of VITEK2 may struggle to detect CST heteroresistance. As a result, VITEK2 may underestimate resistance in certain strains. Manual broth microdilution (BMD) remains the gold standard, as endorsed by the European Committee on Antimicrobial Susceptibility Testing (EUCAST), as it offers greater sensitivity, allowing for precise measurement of MICs, especially at the lower end of the spectrum where VITEK2 may falter, as well as for greater scrutiny and detection of heterogeneity. However, many laboratories lack the resources to perform manual BMD [33].

Ideally, genetic analyses are performed to characterize resistance mechanisms to guide possible synergistic antimicrobial therapy, but rapid molecular diagnostic systems and specialized AST platforms are infrequently available in most clinical laboratories, and outputs from reference laboratories are not always timely when immediate treatment is warranted in highly time-sensitive clinical scenarios [34].

Investigating the synergistic potential of *in vitro* antimicrobial combination therapies may illuminate potential treatment regimens. Synergy testing using gradient-strip MIC determination E-tests is straightforward and rapid, and a means by which the clinical laboratory can provide timely antimicrobial susceptibility data without ready access to highly specialized resources [35]. The principal aim of this study was to evaluate the *in vitro* efficacy of CZA+ATM against a range of CPOs isolated from clinical specimens using E-test-based synergy methods. Secondarily, isolates were tested for susceptibility to FDC, and MICs of CST obtained locally via automated systems were compared with those obtained from the reference laboratory via manual BMD for the same isolate. This was done to appraise the reliability of locally obtained values.

## Methods

### Identification of carbapenemases

Carbapenemases are detected by the microbiology laboratory in St. Vincent’s University Hospital, using Colorex^™^ mSuperCARBA and ertapenem as the screening medium and indicator antimicrobial, respectively, for possible CPEs [36]. Isolates cultured on the selective medium or those producing an ertapenem MIC of ≥0.25 mg l^−1^ on the VITEK2 or a disc diameter <23 mm are highlighted and undergo further testing. For non-Enterobacterales species, decisions on consequent testing are guided by the clinical microbiologist. The Xpert Carba-R allows the rapid identification of the more common carbapenemase genotypes – KPC, NDM, VIM, OXA-48 and IMP – bearing in mind that it is limited to only predetermined genotypes [37]. As such, the modified carbapenem inactivation method (mCIM) is employed as a ‘catch-all’ phenotypic screening modality on suspect isolates, which test negative on Xpert Carba-R, to overcome this limitation [38]. Whilst both testing modalities, GeneXpert Carba-R and mCIM, have been reported to show similar independent sensitivity and specificity [39], mCIM can yield false negative or false positive results depending on the class and level of activity of the carbapenemase, as well as the presence of other mechanisms conferring carbapenem resistance such as efflux pumps or porin loss, as it is a phenotypic test [40]. However, when used in combination, studies have demonstrated that both Xpert Carba-R and mCIM offer very high sensitivity and specificity, especially for detecting rare CPO variants [39]. The presence of concurrent resistance mechanisms is determined using MAST discs [41].

### Tested isolates

All newly detected CPOs are sent to the NCPERLS [4] for genotypic characterization and AST – namely, CST and CZA – for clinical isolates. More specifically, the first isolate per patient that is a confirmed carbapenemase producer from any specimen type, either infection or carriage, is referred, with AST only performed on clinical isolates – defined as those cultured from infection-related specimens such as blood, urine, sputum, bodily fluids or swabs [42]. Each of these isolates is cryopreserved locally.

All referred CPOs from 2017 to 2022 inclusive were reviewed, and those isolated from *clinical* specimens – blood cultures, wound swabs, intra-operative theatre samples, urine and sputum samples – were retrieved for further analysis.

*All* MBLs isolated throughout 2023 from both clinical and rectal screening samples were also analysed to enhance the statistical power of the study. Whilst there is already robust evidence supporting the use of specific agents for SBLs, there is less of a consensus for MBLs, and exploring AST against these organisms is especially important [43,44]. Additionally, given the expected high prevalence of SBLs, particularly OXA-48, within our region [8], this additional selected cohort was included to ensure that a sufficient representative number of MBLs were tested.

### Antimicrobial susceptibility testing

AST for FDC was performed according to EUCAST Disk-Diffusion (Version 10) [45,46].

AST for CST was performed using the VITEK2 system – automated BMD – and local MICs were compared with those attained from the reference laboratory where manual BMD is performed for categorical agreement (CA) and essential agreement (EA) [47,48]. Only isolates cultured from clinical specimens are sent to the reference laboratory for AST for CST. Of note, even though CST MICs are generated locally, they are not reported due to the recognized limitation of the VITEK2 for CST AST, and only those returned from the reference laboratory are reported. As previously mentioned, the VITEK2 system is unreliable for CST AST due to poor accuracy in detecting resistance, non-specific binding of polymyxins to plastic and failure to identify heteroresistance [49]. As a result, it often underestimates MICs, leading to false susceptibility results; BMD remains the gold standard.

MICs to CZA and ATM were determined by gradient-strip MIC determination E-tests (bioMérieux) as per the manufacturer’s instructions.

For the combination CZA+ATM, the E-test strip superposition method was used, which has been extensively validated as an effective method to identify synergistic interactions between antimicrobials [35,50]. An ATM strip was applied for 5 min to an agar plate lawned with a 0.5 McFarland suspension of the test isolate and then removed. A CZA strip was subsequently deposited on the exact place as the prior ATM strip. MICs were read after at least 16 h of incubation at 37 °C, and the fractional inhibitory concentration index (FICI) was calculated – the sum of the quotients of the MIC of the drug combination and the MIC of each drug alone – as previously described [51,52]. For example, if the MIC for CZA and ATM individually was found to be x and y, respectively, and the MIC for CZA+ATM was found to be z, then the FICI is calculated by (z/x)+(z/y). Synergy was defined as FICI ≤0.50, additivity >0.50 to ≤1.00, indifference >1.00 to ≤4.00 and antagonism >4.00.

If an isolate was susceptible to either CZA or ATM on its own, combination therapy was not assessed.

All isolates were tested in duplicate to ensure the reproducibility and reliability of results. *Pseudomonas aeruginosa* ATCC 27853 and *Escherichia coli* ATCC 25922 were used as quality control reference strains.

### Statistical analyses

Descriptive statistics were used to summarize the epidemiology and antimicrobial susceptibility patterns of tested isolates. The chi-square test for independence was applied to assess associations between *β*-lactamase classes and antimicrobial susceptibilities. A *P*-value<0.05 was considered statistically significant.

## Results

Three hundred twenty-two CPOs were isolated throughout the study period. Only 47 clinical isolates and 11 rectal screening isolates were included in the analysis. All others were obtained prior to 2023 from routine rectal surveillance screening swabs in line with local and national IPC policies.

Of these 58 included isolates, 54 (93.1%) were Enterobacterales species and 4 (6.9%) were *Pseudomonas* species. The majority of CPOs were isolated from urine samples (41.4%, *n*=24), with the most common bacteria being *E. coli* (36.2%, *n*=21), *K. pneumoniae* (25.9%, *n*=15) and *Enterobacter cloacae* (22.4%, *n*=13). The microbiological characteristics of all tested isolates are shown in Table 1.

**Table 1.** MICs and categorization of CPOs according to EUCAST clinical breakpoints for tested antimicrobials and the calculated FICIs to determine synergistic effect of CZA+ATM

*Black-, grey- and white coloured MICs correspond to resistant, intermediate and susceptible clinical breakpoints, respectively, according to EUCAST version 13.

na, not applicable; nt, not tested.

OXA-48 was the most commonly detected carbapenemase (37.9%, *n*=22), followed by NDM (34.5%, *n*=20). Co-existing *β*-lactamases of Ambler classes A and C were noted for the majority of CPOs (79.3%, *n*=46), as shown in Table 1.

Twenty-nine isolates (50%) demonstrated susceptibility to FDC. Of the 29 non-susceptible isolates (26 Enterobacterales and 3 *Pseudomonas* species), MBL production was predominant (75.9%, *n*=22). FDC susceptibility was significantly associated with *β*-lactamase class (*P*=0.00038) and Ambler classification (*P*=0.0216). SBL-producing isolates and those classified as Ambler class A exhibited higher susceptibility to FDC compared to MBL-producing or mixed *β*-lactamase isolates harbouring Ambler class B.

According to local AST with the VITEK2, 57 isolates (98.3%) were susceptible to CST. Of the 47 isolates tested by the reference BMD methodology, 44 isolates (93.6%) were susceptible to CST. Complete agreement in categorical breakpoints was noted for 43 isolates, producing a CA of 91.5%. No minor errors were noted, but one major error (ME) with the VITEK2 deeming a ‘susceptible’ isolate (determined by the reference BMD) as ‘resistant’ and three Very MEs (VMEs) with ‘resistant’ isolates being categorized as ‘susceptible’ by VITEK2 MICs. These three organisms, as shown in Table 1, were all dissimilar. Doubling dilution differences revealed an EA of 78.7% within ±1 log_2_ dilution.

CZA+ATM was tested against 26 CPOs, all of which harboured MBLs, with 21 possessing co-existing resistance mechanisms. Synergy was detected for all but one *P. aeruginosa* isolate where additivity was noted, as shown in Table 1. Of the 32 isolates where combination therapy was not assessed, the majority (90.6%, *n*=29) possessed SBLs and were susceptible to CZA monotherapy, whilst three (9.4%) possessed a solitary MBL (NDM) and were susceptible to ATM monotherapy.

## Discussion

CPOs possess the ability to simultaneously produce SBLs and MBLs, in addition to a myriad of other antimicrobial resistance (AMR) genes. This is clearly reflected amongst our cohort, whereby 79.3% (*n*=46) of our CPOs displayed concurrent *β*-lactamases of Ambler classes A and C. Conventional phenotypic AST is often of limited use to clinicians in the setting of these coexisting resistance enzymes, which confer AMR across numerous antimicrobial classes.

Whilst many rapid molecular diagnostic platforms can provide information on the presence of principal resistance genotypes, such as the classification of CPOs by the identification of a target carbapenemase gene, they do not always support the delivery of targeted antimicrobial therapy. Bioinformatics aims to facilitate precise and informed therapeutic decisions, but limited human resources, funding and oversight to support the use of advanced molecular diagnostic systems in the clinical setting pose significant barriers [34]. Consequently, practical methods for the clinical laboratory must be explored to provide meaningful advice to treat these infections in real time.

Traditionally, CST, as an antimicrobial of last resort, remained one of the few viable treatment options for infections caused by CPOs due to limited alternatives. As a polymyxin, it binds to the lipopolysaccharide component of the outer membrane in Gram-negative bacteria, leading to destabilization and eventual cell death – a unique mechanism which allows it to overcome enzymatic resistance mechanisms displayed by CPOs. However, CST is known to be particularly nephrotoxic and neurotoxic, which can be potentially irreversible. So close monitoring, prompt intervention and individualized management are vital to mitigate the risks and maximize the chances of recovery [32].

The development of CST AMR was of particular global concern as limited therapeutic options for difficult-to-treat infections were now becoming more limited. Systematic reviews found that CST AMR amongst Enterobacterales has been gradually increasing over prior decades, with rates now being reported as high as 60% in Asia and 25% in Europe – all attributed to the dissemination of *mcr*-containing plasmids, which facilitate modification of lipid A in the cell membrane [53,55].

As such, AST in clinical laboratories is warranted to allow effective management, treatment and surveillance monitoring. The time-consuming, cumbersome and resource-intensive BMD method endorsed by EUCAST is difficult to implement in the routine laboratory. Commercial automated systems like the VITEK2 are more commonplace with their streamlined set-up and automated reading and interpretation. However, CST MICs are deemed unreliable through automated systems due to issues with induced heteroresistance and low-level resistance [33]. Comparative studies found VME rates well in excess of the 1.5% rate recommended by the Clinical and Laboratory Standards Institute, with some institutions reporting up to 86% discordance [33,56, 57]. Although a CA of 91.5% was found amongst our isolates, EA was 78.7%, and a VME rate of 6.3% was reported. Therefore, the VITEK2 may provide presumptive categorical information, but MICs must be confirmed by BMD as misinterpreting CST AST results can lead to treatment failures and even mortality in the setting of VMEs.

Another hopeful agent in the fight against CPOs is FDC. Based on promising findings from the CREDIBLE-CR and APEKS studies, FDC has been approved for the treatment of infections caused by aerobic Gram-negative organisms in adults in Europe and the USA. It is even included as a therapeutic option in the recent guidance on the treatment of antimicrobial-resistant Gram-negative infections published by the Infectious Diseases Society of America [26,28,43]. Numerous studies have since evaluated the *in vitro* activity of FDC against a variety of bacteria, and it appears to perform favourably against CPOs harbouring SBLs, but not MBLs [58,59].

An analysis of 305 Enterobacterales and 111 *P. aeruginosa* isolates from Public Health England’s Antimicrobial Resistance and Healthcare Associated Infections Reference Unit found that isolates harbouring NDM carbapenemases were more frequently non-susceptible to FDC, at an AMR rate of 59% [60]. A comparable AMR rate of 52% for NDM-producing CPOs was reported amongst French and Belgian isolates, whilst Taiwan and the USA quote 22% and 17% AMR specifically for NDM-producing CPOs, respectively [58]. For CPOs with SBLs and non-NDM MBLs, all the aforementioned studies report FDC susceptibility >90%. Our study found a much lower susceptibility rate of 50% amongst all CPOs, with a resistance predominantly identified in those expressing MBLs (75.9%, *n*=22–17 NDM, 3 IMP and 2 VIM strains).

Differences in AST may account for this variability, as the abovementioned studies performed BMD whilst the disc diffusion methodology was adopted locally. Whilst BMD remains the reference AST methodology for FDC, it is not commonly performed due to its complexity, technical challenges and the need for iron-depleted conditions to ensure accurate results. The specialized media and stringent quality control requirements make BMD labour- and resource-intensive and impractical for routine testing. In contrast, disc diffusion provides a standardized, cost-effective and reproducible alternative, endorsed by regulatory guidelines. Disc diffusion offers a practical method for routine laboratories to assess FDC AST whilst maintaining accuracy and clinical relevance [46,61].

Moreover, targeted antimicrobial combination therapy against different AMR mechanisms can be selected with knowledge of the pathogen-specific genotype. This is best illustrated by the use of CZA+ATM for MBL-producing CPOs; for even though MBLs fail to hydrolyse ATM, a monocyclic *β*-lactam, the various coenzymes – oxacillinases, AmpC and ESBLs – which are frequently co-expressed by the MBL plasmids, can hydrolyse ATM and render it ineffective. As such, it stands to reason that if the co-enzymes are inhibited, ATM is then free to manifest its bactericidal activity. AVI, a novel diazabicyclooctane *β*-lactamase inhibitor, displays potent *in vitro* activity against most non-MBL enzymes – including Ambler classes A, C and D – in order to restore the activity of ATM. AVI is only currently commercially available as CZA, and whilst CZA monotherapy is ineffective due to CTZ being readily hydrolysed by the MBL, the combination of CZA+ATM has been reported with significant success, tried and tested through recognition and understanding of the various AMR mechanisms present [29,31].

ATM susceptibility was fully restored for 86% and 89% of MBL-producing Enterobacterales when combined with CZA in a French and American study, respectively [47,62]. Similarly, CZA+ATM demonstrated *in vitro* activity against 81% of Enterobacterales possessing MBLs [63], whilst another report found 90% synergy and 10% additivity amongst a selection of CPOs from the Antimicrobial Resistance Isolate Bank in the USA [29]. Synergy was also detected amongst a collection of CST-resistant *K. pneumoniae* isolates recovered from clinical samples in France, Colombia and Turkey [64], and an Iranian study found a synergistic effect in 98.8% and 95% of pan-drug-resistant *Klebsiella* species and *Escherichia coli* isolates, respectively [65].

Our study adds to this growing body of literature regarding CZA+ATM as a therapeutic agent for MBL-producing CPOs harbouring co-enzymes, with synergy detected against 96.2% (*n*=25) of our tested isolates, and additivity noted in the remaining isolate.

Whilst initial *in vitro* studies demonstrated the synergistic potential of CZA+ATM against CPOs, these findings have now been reinforced by clinical studies, where real-world outcomes consistently show improved survival, bacterial eradication and reduced clinical failure compared to traditional treatment regimens.

In Spain, a retrospective analysis of 24 patients with VIM-producing Enterobacterales and *P. aeruginosa* infections reported a 30-day mortality rate of 17% and a clinical failure rate at day 14 of 8% with CZA+ATM [66]. Similarly, an Italian study of 40 patients with bloodstream infections caused by NDM-producing Enterobacterales found that 10 of 12 patients treated with CZA+ATM survived, whilst the two who died had severe comorbidities, suggesting that mortality was more closely linked to underlying conditions rather than treatment failure [67]. In Poland, 23 patients with infections caused by NDM-producing *K. pneumoniae* achieved bacterial eradication following treatment with CZA+ATM, though four died due to non-infection-related causes [68].

Further strengthening these findings, a prospective observational study conducted across three hospitals in Italy and Greece enrolled 102 patients with bloodstream infections caused by MBL-producing Enterobacterales and compared CZA+ATM to alternative antimicrobials. The combination therapy group had a significantly lower 30-day mortality rate (19.2% vs. 44%, *P*=0.007), reduced clinical failure at day 14 (HR 0.30; *P*=0.002) and shorter hospital stays (HR 0.49; *P*=0.007), reinforcing its role as a preferred treatment option [69]. Smaller prospective case series have also documented comparable successful outcomes [70,71].

This enhanced activity observed with CZA+ATM, compared to either agent alone, is promising, especially in light of diminishing treatment options for CPOs. Furthermore, it highlights that understanding the intricacies of AMR mechanisms is vital in elucidating combination therapies and reinforces the need to equip clinical laboratories with the necessary tools to allow clinicians to make informed and timely therapeutic decisions.

With this in mind, antimicrobial stewardship programmes (ASPs) remain essential in balancing this innovation with sustainability, ensuring that novel therapies remain effective in the long term [72]. As novel antimicrobial agents are introduced, their clinical utility depends on careful stewardship to prevent premature resistance development. ASPs achieve this by promoting evidence-based prescribing, optimizing dosing strategies and integrating rapid diagnostics to ensure targeted therapy. Additionally, they play a key role in surveillance, monitoring resistance patterns and guiding empiric treatment strategies to minimize unnecessary broad-spectrum antimicrobial use [72,73]. By aligning therapeutic advancements with responsible antimicrobial management, ASPs not only enhance patient outcomes but also safeguard the longevity of treatment options in the fight against multidrug-resistant infections, such as those from CPOs.

Whilst data demonstrates the efficacy of FDC and CZA+ATM against infections caused by CPOs, cost considerations must be taken into account when evaluating their clinical utility. These agents are more expensive than traditional carbapenems or polymyxins, raising concerns about affordability, particularly in resource-limited healthcare settings. However, their efficacy may lead to shorter hospital stays, reduced need for intensive care and lower overall healthcare costs associated with treatment failure and complications from suboptimal therapies. Studies from the USA [74], Iran [75] and Peru [76] have assessed the cost-effectiveness of CZA+ATM for infections caused by CPOs using decision-analytic models incorporating quality-adjusted life years and incremental cost-utility ratios. These analyses support the economic and clinical viability of CZA+ATM, demonstrating that whilst its initial cost is higher than conventional therapies, its use is associated with lower overall healthcare expenditures.

Our study is limited by its small sample size of CPOs from a very discrete patient population. As we only included clinical isolates from 2017 to 2022 and then only MBL isolates in 2023, our data does not reflect the true epidemiology of CPOs at our institution. Continued validation of laboratory testing methodologies should be performed on a larger sample of genotypically diverse pathogens. Additionally, our study was not designed to include *in vivo* data to validate the safety and efficacy of the treatment strategies in clinical settings, which limits the direct applicability of our findings to real-world scenarios; this represents a key area for future translational research.

## Conclusion

FDC appears to perform favourably against CPOs harbouring SBLs, but not MBLs. The VITEK2 may provide presumptive categorical information for CST susceptibility, but MICs must be confirmed by BMD as VMEs can lead to inexplicable treatment failures and even mortality. Most importantly, our study confirms the potent *in vitro* activity of CZA+ATM against CPOs expressing multiple *β*-lactamases.

## Abbreviations

AMR: antimicrobial resistance
ASPs: antimicrobial stewardship programmes
AST: antimicrobial susceptibility testing
ATM: aztreonam
AVI: avibactam
BMD: broth microdilution
CA: categorical agreement
CPE: carbapenemase-producing Enterobacterales
CPOs: carbapenemase-producing organisms
CRE: carbapenem-resistant Enterobacterales
CST: colistin
CTZ: ceftazidime
CZA: ceftazidime-avibactam
EA: essential agreement
ESBLs: extended-spectrum beta-lactamases
EUCAST: European Committee on Antimicrobial Susceptibility Testing
FDC: cefiderocol
FICI: fractional inhibitory concentration index
IMP: integron-associated MBL
IPC: infection prevention and control
KPC: *Klebsiella pneumoniae* carbapenemase
MBL: metallo-*β*-lactamase
mCIM: modified carbapenem inactivation method
MDRKP: multi-drug-resistant *Klebsiella pneumoniae*
ME: major error
NCPERLS: National Carbapenemase-Producing Enterobacterales Reference Laboratory Service
NDM: New Delhi metallo-*β*-lactamase
SBLs: serine-*β*-lactamases
VIM: Verona integron-encoded MBL
VMEs: very major errors
VMEs: very major errors
